# Alterations of *Candidatus* Liberibacter asiaticus-Associated Microbiota Decrease Survival of *Ca.* L. asiaticus in *in vitro* Assays

**DOI:** 10.3389/fmicb.2018.03089

**Published:** 2018-12-21

**Authors:** Kazuki Fujiwara, Toru Iwanami, Takashi Fujikawa

**Affiliations:** ^1^Kyushu Okinawa Agricultural Research Center, National Agriculture and Food Research Organization, Koshi, Japan; ^2^Institute of Fruit Tree and Tea Science, National Agriculture and Food Research Organization, Tsukuba, Japan

**Keywords:** *Candidatus* Liberibacter asiaticus, huanglongbing, *Candidatus* Liberibacter asiaticus-associated microbiota, culture medium, RNA sequence, metagenomics

## Abstract

Phloem-inhabiting bacterial phytopathogens often have smaller genomes than other bacterial phytopathogens. It is thought that they depend on both other phloem microbiota and phloem nutrients for colonization of the host. However, the mechanism underlying associations between phloem-inhabiting phytopathogens and other phloem microbiota are poorly understood. Here, we demonstrate that the survival of *Candidatu*s Liberibacter asiaticus (CLas), a cause of huanglongbing (citrus greening disease), depends on interplay with a specific subset of CLas-associated microbiota. CLas was not susceptible to oxytetracycline *in vitro*. However, oxytetracycline treatment eliminated a particular sub-community dominated by the *Comamonadaceae*, *Flavobacteriaceae*, *Microbacteriaceae*, and *Pseudomonadaceae*, decreasing CLas survival. We speculate that CLas uses ecological services derived from CLas-associated microbiota to colonize the host and to construct a pathogen-associated community that stimulates disease development.

## Introduction

Plant microbiota are major determinants of plant health. Substantial progress has been made in understanding the community structures and functions of phyllosphere and rhizosphere microbiota that confer microbial ecosystem services such as nutrient supply, growth promotion, and tolerance to stresses (Vorholt, 2011; Mendes et al., 2013). Changes to microbiota structure lead to changes in the structural and functional maturity of services (Weller et al., 2002). Phloem-associated microbiota constitute an endogenous ecosystem adapted to the phloem environment (Trivedi et al., 2016; Bendix and Lewis, 2018). Phloem-limited phytopathogenic bacteria (e.g., *Candidatus* Phytoplasma, *Candidatus* Liberibacter, and genus *Spiroplasma*) are systemically distributed throughout plants (Perilla-Henao and Casteel, 2016). Interplay between such pathogens and other phloem-associated microbiota is thought to trigger structural and functional changes in phloem ecosystems that contribute to pathogenic colonization (Trivedi et al., 2016).

*Candidatu*s Liberibacter asiaticus (CLas) is the causal agent of huanglongbing (citrus greening disease), which causes devastating yield losses in citrus worldwide (Garnier et al., 2000; Bové et al., 2006). Antibiotics are effective at eliminating CLas and are used for its control; beta-lactam and tetracycline antibiotics have been broadly applied via several techniques, including foliar spray, trunk injection, and root drench (Zhang et al., 2010, Zhang M. et al., 2011; Yang et al., 2015). However, it is unclear whether antibiotic treatment is always feasible for the control of CLas in practice. Recent studies have demonstrated that antibiotic treatment induced significant changes in the microbial community structure in citrus leaves and indicated the action of CLas-associated microbiota in establishing habitability for CLas in the phloem (Zhang et al., 2013b; Blaustein et al., 2017). However, the underlying mechanisms have not yet been clarified.

Here, we investigated potential roles of CLas-associated microbiota in the habitability of the phloem for CLas, First, we developed a new culture medium that allowed us to reveal the response of CLas strain Ishi-1 to antibiotic stress in an *in vitro* culture assay. Second, we disrupted CLas-associated microbiota extracted from CLas-infected leaves with an antibiotic *in vitro* and performed a metagenomic analysis to evaluate community structural changes associated with habitability for CLas.

## Materials and Methods

### KEGG Pathway Analysis

We compared pathways among CLas and 23 closely related species of α-Proteobacteria using data from the Kyoto Encyclopedia of Genes and Genomes (KEGG) database^1^. At the second level in the KEGG module hierarchy under metabolic pathways, when we found a candidate species in a reference module, the conservation level in the defined pathway was classified as having a full, a partial, or no functional unit. Functional units not relevant to or weakly associated with CLas (carbon fixation; energy metabolism from nitrogen, methane, and sulfur; and sterol biosynthesis) were excluded. In the analysis of gene functions, we classified genes into the second level in the KEGG Orthology hierarchy. Genes without KEGG Orthology assignment were labeled by their biological features and correspondence to the relevant pathway.

### Inoculum Preparation for Culture

We prepared culture inoculum from citrus trees infected with CLas (strains Ishi-1 and Miyako-13) (Furuya et al., 2010; Tomimura et al., 2010), which are maintained in a quarantined greenhouse under a special permit (26Y1214) issued by the Ministry of Agriculture, Forestry and Fisheries. CLas infection status was confirmed by both the development of foliar disease symptoms and the presence of CLas determined by PCR as described (Fujikawa et al., 2013). CLas-infected leaves were surface-sterilized by wiping 3 times with 70% ethanol. About 0.05 (± 0.01 SD) g of leaf midribs were cut and finely chopped, homogenized in 400 μL sterilized distilled water in a BioMasher III mini-homogenizer tube (Nippi, Tokyo, Japan), and centrifuged for 2 min at 7000 ×*g*. After centrifugation, 400 μL of flow-through solution was discarded and the precipitate was resuspended in 400 μL of sterilized distilled water. This suspension was used as the culture inoculum. Inoculum was also prepared from CLas-free (healthy) leaves.

### Culture and Observations

CLas strain Ishi-1 was grown in our new culture medium (Supplementary Table S1) supplemented with cycloheximide (30 ppm) and ampicillin (50 ppm). The medium was autoclaved before the antibiotics were added, and its pH was adjusted to around 7 with NaOH. For plate culture, 100 μL of inoculum was applied to each of three plates, which were then incubated for 2 months at 25°C. Three 1-cm^2^ squares of agar from each plate were cut out for DNA extraction and grouped as one biological replicate. For liquid culture, 50 μL of inoculum was added into 5 mL of culture medium in each of nine test tubes, which were then incubated for 2 months at 25°C on a shaker (60 rpm). From each replicate, 50 μL of culture was collected for DNA extraction with a DNeasy Plant Mini Kit (Qiagen, Valencia, CA, United States) according to the manufacturer’s instructions. To confirm detection of DNA as a result of Ishi-1 proliferation, we used reference DNA for comparison. Template DNA was prepared from PCR amplicons of CLas 16S rDNA. The amount of template DNA was adjusted to the DNA content in 10^5^ cells/mL with liquid culture medium, and 100 μL of the adjusted template DNA was applied to each of three plates, which were then incubated for a month at 25°C. Three 1-cm^2^ squares of agar from each plate were cut out for DNA extraction and grouped as one biological replicate. CLas and template DNA were quantified by SYBR Green real-time PCR using the Las931/LSS primer set (Supplementary Table S2).

Microscope slides were observed under an Axioplan 2 epifluorescent microscope (Carl Zeiss, Jena, Germany) with a Plan-Apochromat 40 × /0.95 corr. (Carl Zeiss) and a Plan-Apochromat 100 × /1.4 oil objective (Carl Zeiss). Fluorescence *in situ* hybridization assay using an RNA-targeting probe was performed as described (Fujikawa et al., 2013). Liquid culture medium was observed under a TM4000 scanning electron microscope (SEM) (HITACHI, Tokyo, Japan). For sample preparation, 2 mL of the cultured sample was centrifuged for 5 min at 10000 × *g* and 2 mL of flow-through solution was discarded. The precipitate was resuspended and fixed in 1 mL of 0.1 M phosphate-buffered saline (PBS) supplemented with 2% paraformaldehyde for 2 h. The fixed sample was centrifuged for 5 min at 10000 ×*g* and the precipitate was resuspended in 1 mL of 0.1 M PBS. For coating, 30 μL of the suspension was applied onto a nano-percolator (JEOL, Tokyo, Japan) which was coated with a thin gold layer using a JEE-400 vacuum evaporator (JEOL), followed by applying a TI-Blue (platinum-Blue) solution (Nisshin EM Co., Ltd., Tokyo, Japan) onto the nano-percolator. The staining was kept for an hour. The stained sample was rinsed with sterilized distilled water and syringe-vacuumed. This step was repeated three times. The prepared sample with the nano-percolator was used for SEM observation.

### *In vitro* Assays With Antibiotics and Carbohydrates

Each of eight antibiotics (ampicillin, chloramphenicol, kanamycin, oxytetracycline, polymyxin B, rifampicin, streptomycin, and tetracycline) was tested at three concentrations (50, 500, and 1000 ppm) in plate culture. Alternative culture plates excluding four carbohydrate sources (glucose, fructose, sucrose, and starch) were also prepared. The osmolality of the culture medium was determined by the freezing point depression method on an Auto & Stat OM-6030 osmometer (Arkray Inc., Kyoto, Japan). Plates were prepared in triplicate and incubated for 2 weeks at 25°C. Three 1-cm^2^ squares were cut from each plate for DNA extraction and grouped as one biological replicate. CLas was quantified by SYBR Green real-time PCR.

### CLas Ishi-1 Incubation With CLas-Associated Microbiota

CLas-infected leaves were surface-sterilized by wiping 3 times with 70% ethanol and immersed in 70% ethanol for 1 min. Midribs of 18 Ishi-1-infected leaves were cut out and chopped into 2–3-mm pieces. The chopped midribs were all mixed and then divided into two samples (equivalent to 9 leaves/sample). Each sample was ground in 3.6 mL of either sterilized distilled water or 1000 ppm oxytetracycline (400 μL/leaf) with a mortar and pestle and then incubated in those solutions for 4 h at 25°C under plastic wrap. Each treated sample was transferred into a BioMasher III spin centrifuge tube filter and centrifuged for 2 min at 7000 ×*g*. After centrifugation, the flow-through solution was discarded, and the precipitate was resuspended in 3.6 mL of sterilized distilled water. This washing step was performed three times. Precipitates from each treatment were resuspended in 1.8 mL of sterilized distilled water (200 μL/leaf). Each solution was divided into nine samples (equivalent to 1 leaf/sample) in plastic test tubes, which were then incubated at 25°C. Three samples were collected at each of 0, 90, and 120 h. CLas was quantified by TaqMan probe real-time PCR. Experiments were also performed using CLas-free (healthy) leaves. Metagenomic analysis was carried out using a representative sample which was prepared by mixing three samples of extracted DNA (0 h) in each treatment.

### Conventional and Real-Time PCR

CLas was detected by conventional PCR as described (Fujikawa et al., 2013) with 45 cycles of amplification using CLas-specific primers (Fujikawa and Iwanami, 2012) and the new CLas primer sets Las931/LSS, mota-f/mota-r, and murg-f/murg-r(Supplementary Table S2). CLas was quantified by real-time PCR using both SYBR Green and fluorescent probe–based methods. SYBR Green real-time PCR was performed using the primer set Las931/LSS. To label and amplify templates, SYBR Premix Ex Taq II (Tli RNase H Plus; Takara, Shiga, Japan) was used according to the manufacturer’s protocols with modifications, under the following PCR conditions: pre-denaturation at 96°C for 10 min; 45 cycles of denaturation at 96°C for 30 s, annealing at 55°C for 30 s, and extension at 72°C for 30 s; further denaturation at 96°C for 15 s; holding at 55°C for 1 min; and heating from 55 to 96°C for 15 s for melting curve analysis. CLas cell concentrations were calculated from the relation between quantity and molarity of CLas 16S rDNA (Degen et al., 2006). The amount of template DNA was adjusted to the DNA content in 10^8^ cells/μL, and a 10-fold dilution series was prepared for standard curve analysis (*y* = -1.513*x* ++ 33.806, *R*^2^ = 1). For fluorescent probe–based methods, TaqMan probe real-time PCR was performed as described (Li et al., 2006) using the HLBas/HLBr/HLBp primer–probe set.

### RNA Sequences and Analysis

Culture medium was prepared with or without 1000 ppm oxytetracycline. Four plates were prepared for each condition. For culture, 100 μL of inoculum was applied to each plate and then incubated for 2 weeks at 25°C. A culture sample was collected from each plate and used for RNA extraction with an RNeasy Plant Mini Kit (Qiagen). Following the manufacturer’s protocols, templates were created from sample RNAs with an Ion Total RNA-Seq kit v. 2 (Thermo Fisher Scientific Inc., Waltham, MA, United States) and an Ion PGM Hi-Q OT2 kit on an Ion OneTouch 2 system (Thermo Fisher Scientific). The templates were sequenced with an Ion PGM Hi-Q sequencing kit and a 318 Chip kit v. 2 on an Ion PGM next generation sequencer (Thermo Fisher Scientific). Sequence data were assembled and analyzed in CLC Genomic Workbench software (CLC Bio, Qiagen). The genome sequence of Ishi-1 (GenBank acc. no. AP014595) was used as the reference for RNA-Seq mapping and assembly of sequence reads. The gene expression value was calculated from RPKM (Reads Per Kilobase per Million mapped reads). Differential expression analysis used log_2_(*x* + 0.0001) data. Principal coordinates analysis based on differential gene expression profiles was performed in the EdgeR package of R v. 3.3.0 software.

### 16S Metagenomics Sequencing and Analysis

16S metagenomics templates were generated from extracted DNA using primers designed to target the V3 and V4 regions of the 16S rRNA gene sequence with an Ion 16S Metagenomic Kit (Thermo Fisher Scientific) and an Ion PGM Hi-Q OT2 kit on an Ion OneTouch 2 system. The templates were sequenced with an Ion PGM Hi-Q sequencing kit and a 318 Chip v. 2 on an Ion PGM next generation sequencer. Sequence data were analyzed in CLC Genomics Workbench software with the Microbial Genomics Module. GreenGenes data (greengenes.lbl.gov) were used as references for microbial targets.

### Data Availability

Raw sequencing data generated for transcriptomic and metagenomic projects were deposited in the NCBI Short Read Archive (SRA) database with accession numbers SRP110876, SRP110878, SRP110882, and SRP110883.

## Results

### KEGG Analysis for CLas

**FIGURE 1:**
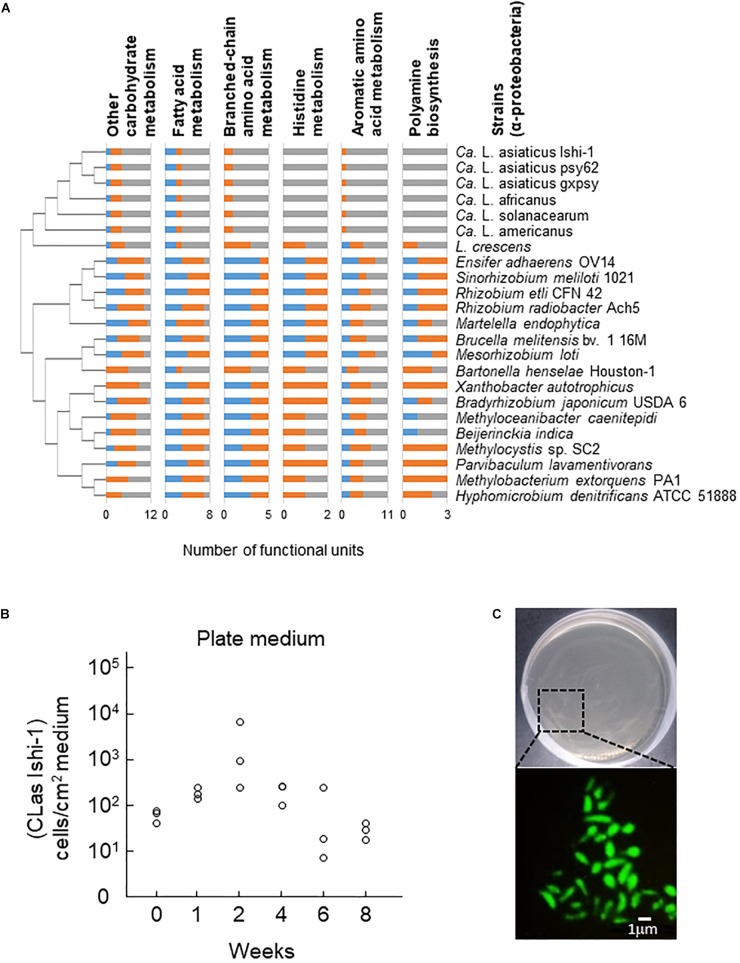
Culture of *Candidatus* Liberibacter asiaticus (CLas) *in vitro*. **(A)** Metabolic pathway analysis of 23 representative species of α-Proteobacteria. Less-conserved metabolic pathways in *Candidatus* Liberibacter species (>50% dissimilarity to other α-Proteobacterial species) are shown. Blue, full functional unit; orange, partial functional unit; gray, no functional unit. Phylogenetic relationships were analyzed on the basis of nucleotide sequences of 16S ribosomal DNA from GenBank by the neighbor-joining method in MEGA 6 software. Stability was assessed using 1000 bootstrap replications. **(B)** CLas Ishi-1 was grown in newly developed culture medium supplemented with ampicillin (50 ppm). Ishi-1 was quantified by real-time PCR using DNA templates prepared from 1-cm^2^ pieces of agar (*n* = 3, each value plotted individually). **(C)** Microscopic observation of CLas Ishi-1 by fluorescence *in situ* hybridization (with Las-specific probe LSS) using samples incubated for 2 weeks.

CLas has a very small genome (1200 to 1300 kbp) and lacks functional metabolic pathways (Duan et al., 2009; Katoh et al., 2014; Zheng et al., 2015), indicating a host-dependent life cycle. When we explored metabolic pathways specifically deleted in CLas, the cluster of *Candidatus* Liberibacter species lacked most genes responsible for the metabolism of carbohydrates, fatty acids, several amino acids, the aromatics, and secondary metabolites in comparison with other α-Proteobacteria (Supplementary Figures S1–S3). In particular, it had few genes for other carbohydrate biosynthesis (involved in nucleotide sugar biosynthesis), fatty acid biosynthesis (involved in acetyl-CoA synthesis), or amino acid metabolism (including the biosynthesis of the branched-chain amino acids valine and isoleucine, and of aromatic amino acids tryptophan, phenylalanine, and tyrosine); and no genes for histidine metabolism or polyamine biosynthesis (involved in spermidine biosynthesis), except for *Liberibacter crescens*, the only known culturable species (Figure 1A). These findings are consistent with a recent report that an unculturable mutant of *L. crescens* lacks genes responsible for the biosynthesis of fatty acids, aromatic amino acids, histidine, cysteine, lipopolysaccharides, and several vitamins (Lai et al., 2016). Although little is known about the extent to which unculturable *Liberibacter* species depend on these molecules, differences in metabolism between culturable and unculturable *Liberibacter* species indicate potential metabolic pathways that are required for CLas survival in culture.

### CLas Culture *in vitro*

Previous studies established that application of nutrients extracted from citrus tissues was effective for culturing CLas (Sechler et al., 2009; Parker et al., 2014). However, as it is difficult to prepare identical plant extracts for culture, we devised a new culture medium with 16 nutrients that we selected on the basis of KEGG analysis (Supplementary Table S1). Both plate and liquid media supported the proliferation of CLas strain Ishi-1 (Figure 1B and Supplementary Figure S4A). Reference DNA of Ishi-1 incubated on culture plates decreased over time (Supplementary Figure S4B), suggesting that proliferation of Ishi-1 observed in culture was derived from Ishi-1 cells grown in culture rather than dead cells or variation in cell density. The serial transfer of 50 μL of liquid cultures into 5 mL of new medium was performed, and viability was maintained even after two serial passages with culture duration of a month. Detection of CLas was also confirmed by different candidate primers targeting 16S rRNA genes, flagellar motor protein gene *motA*, and lipid II flippase gene *murG* (Supplementary Figure S4C). Generation time, calculated from an initial density of 756 (± 589 SD) cells/mL and a final density of 5418 (± 2796) cells/mL after 2 weeks in liquid medium, was 118 h. Contamination prevented the growth of CLas in medium without antibiotics (Supplementary Figure S5). The osmolality of the culture medium was 312 mOsm, within the range of phloem solution (240–600 mOsm) (Whitcomb and Tully, 1979). Importantly, culture medium excluding four carbohydrates (glucose, fructose, sucrose, and starch), which are major nutrients in the phloem (Hijaz and Killiny, 2014), poorly supported the growth of CLas (Supplementary Figure S6), although its osmolality was 286 mOsm. This difference indicates that carbohydrates play a key role in supplying energy for the growth of CLas.

CLas strain Miyako-13 struggled to grow in culture (Supplementary Figure S7). Ishi-1 lacks prophage regions in its genome, which other CLas strains, such as Miyako-13, use for cell lysis in plant infections (Zhang S. et al., 2011). This difference suggests that the presence of the prophages in Miyako-13 may lead to bacterial cell lysis in culture, as *in planta*. Collectively, these results demonstrate that our new culture medium can culture strain Ishi-1 *in vitro*.

### Microscopic Observations

Tiny dot-like patterns appeared on the surface of the plate medium a week after the start of incubation, but they were not CLas colonies. Microscopic and SEM observations revealed the presence of CLas-like cells in plate culture at 2 weeks and elongated cells in liquid culture at 4 weeks (Supplementary Figure S8). Fluorescent *in situ* hybridization revealed Ishi-1 cells in plate culture, indicating cell viability (Figure 1C). In liquid culture, it was difficult to morphologically differentiate Ishi-1 cells from cultured substances such as starch-like substances by fluorescence microscopy because the cultured substances fluoresce, and their aggregation with Ishi-1 often masked the Ishi-1-specific fluorescence. In SEM observation, Ishi-1 cells were the presence of Ishi-1 in culture was observed only when we used an inoculum prepared from CLas-infected leaves.

### Response of CLas Ishi-1 to Antibiotic Stress in Culture

**FIGURE 2:**
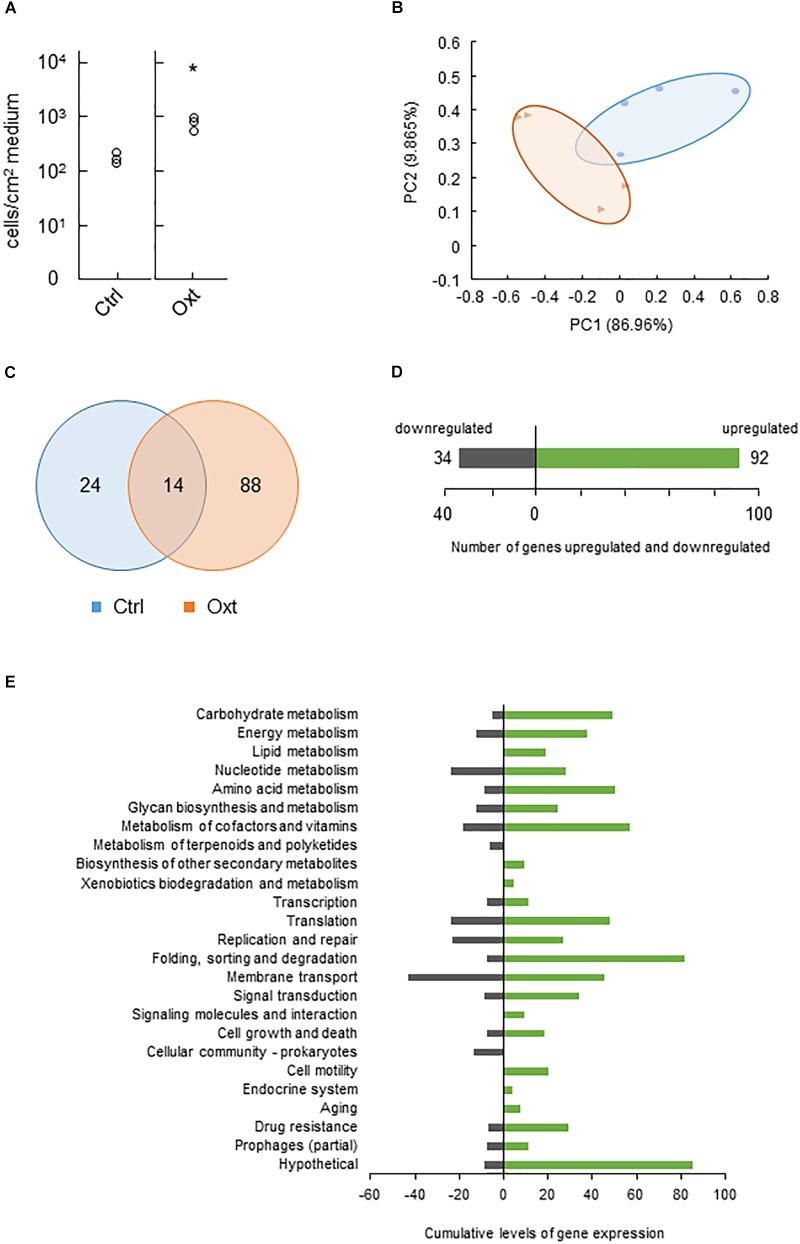
Functional changes of CLas in response to oxytetracycline stress. **(A)** Growth of CLas Ishi-1 was promoted at 2 weeks in culture medium supplemented with oxytetracycline (Oxt) at 1000 ppm. Ishi-1 was quantified by real-time PCR using DNA templates prepared from 1-cm^2^ pieces of agar (*n* = 3, each value plotted individually). Control (Ctrl) was prepared without Oxt. Proliferation was significantly higher with Oxt (*n* = 3, ^∗^*P* < 0.05, Student’s *t*-test). **(B)** Principal coordinates analysis plot based on differential gene expression profiles between Ctrl (•) and Oxt (▲). PC1 and PC2 explain 86.96 and 9.865%, respectively, of the distribution. RNA of Ishi-1 was sequenced using total RNAs extracted from Ctrl and Oxt plates reported in panel **A** with four plate replicates each. **(C)** Numbers of Ishi-1 genes expressed in Ctrl and Oxt. The gene expression value was averaged; the total number of genes expressed in each treatment was shown. **(D)** Numbers of Ishi-1 genes upregulated (green) and downregulated (gray) in Oxt relative to Ctrl. **(E)** Changes in gene expression induced by Oxt. Differentially expressed genes were classified into KEGG pathways; cumulative levels of gene expression in each pathway are indicated by bar lengths.

We next tested CLas behaviors in response to antibiotic stress *in vitro*. Assays using eight antibiotics demonstrated that most of them, except the higher concentrations of polymyxin B, were unlikely to inhibit Ishi-1 even at the highest concentration (1000 ppm) during incubation for 2 weeks (Supplementary Figure S9). Instead, the growth of Ishi-1 was significantly enhanced in the presence of 1000 ppm oxytetracycline (Figure 2A). These results show that oxytetracycline was likely to accelerate the growth of CLas in culture.

Resistance to tetracycline or oxytetracycline is often attributable to the presence in bacteria of resistance genes encoding efflux pumps, ribosomal protection proteins, and inactivating enzymes (van Hoek et al., 2011). To explore the mechanism of Ishi-1’s response to oxytetracycline, we sequenced RNAs to analyze genome-wide gene expression. The sequencing generated 601 390 reads from oxytetracycline treatment and 457 126 reads from the control, which we aligned to the reference sequence of CLas Ishi-1. Approximately 14% of the oxytetracycline reads and 23% of the control reads were uniquely mapped and quantified from RPKM. One-sixth of reads constituted citrus genes. Since the remaining read sequences were not unique, they were excluded from the analysis. Exposure to oxytetracycline caused differential gene regulation (Figure 2B), with treatment-specific regulation of 112 genes (nearly 10.2% of all genes), of which 88 were unique to oxytetracycline (Figure 2C). In total, 92 genes were upregulated (3 significantly; Supplementary Table S3) and 34 genes downregulated (Figure 2D). The transcriptome profiles revealed no homologs of tetracycline resistance genes such as *tet*, *otr*, and *tcr*. However, several genes related to drug resistance or membrane transport were upregulated (Figure 2E and Supplementary Table S4), as previously reported for multidrug resistance (Munita and Arias, 2016), one gene considerably so: CGUJ_01130, which encodes proline/glycine betaine ABC transporter permease. Hence, although oxytetracycline-specific efflux is unlikely, multidrug efflux may indicate other resistance mechanisms in Ishi-1.

### CLas Ishi-1 Incubation With CLas-Associated Microbiota *in vitro*

**FIGURE 3:**
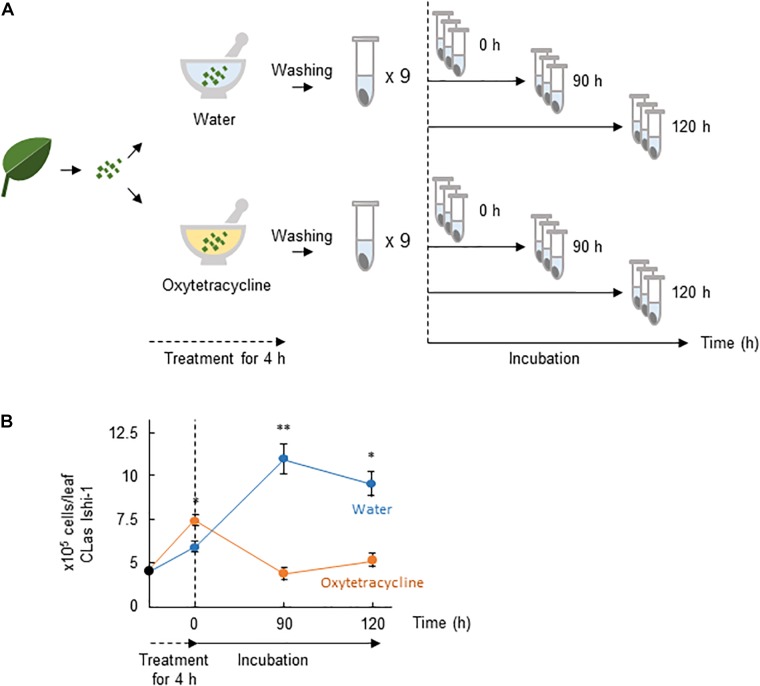
Oxytetracycline treatment alters the growth conditions provided by CLas-associated microbiota for CLas. **(A)** Procedures for incubation of CLas Ishi-1 among CLas-associated microbiota. CLas-infected plant materials were ground in oxytetracycline or sterilized distilled water and held for 4 h at 25°C. Each was rinsed three times and resuspended in sterilized distilled water, in which it was incubated for up to 120 h. **(B)** CLas Ishi-1 was not eradicated by oxytetracycline among CLas-associated microbiota but its growth suffered after the removal of oxytetracycline. CLas cells were quantified by real-time PCR of total DNA extracted from each sample. Data are means ± SD (*n* = 3). Statistical significance was determined by Student’s *t*-test (^∗^*P* < 0.05, ^∗∗^*P* < 0.01).

The presence of CLas-associated microbiota in infected citrus plants is thought to facilitate the survival of CLas and the development of huanglongbing (Blaustein et al., 2017). We thus postulated that microbial ecosystems play a critical role in creating habitability for CLas, and that oxytetracycline disrupts the ecosystem. To test this hypothesis, we investigated the effect of oxytetracycline on survival of Ishi-1 mixed with CLas-associated microbiota. It did not eradicate Ishi-1 in the presence of CLas-associated microbiota (Figures 3A,B). However, subsequent incubation in sterilized distilled water showed a significant decrease in CLas density at 90 h (*P* < 0.01) and 120 h (*P* < 0.05) in the oxytetracycline-treated community (Figure 3B). The results show that oxytetracycline treatment altered the growth conditions of the CLas-associated microbiota, leading to inhibition of the growth of CLas.

### Metagenomic Analysis of CLas-Associated Microbiota *in vitro*

**FIGURE 4:**
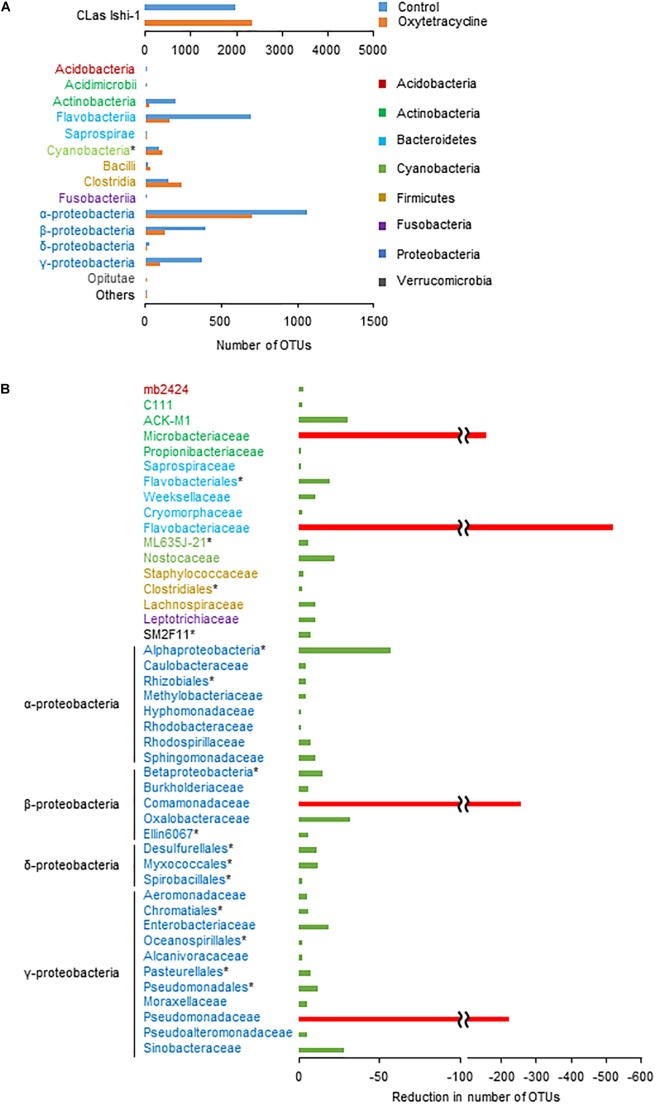
Oxytetracycline-triggered structural changes in phloem-associated microbiota. **(A)** Absolute numbers of dominant OTUs at class or ^∗^higher rank in oxytetracycline and water treatments at 0 h. **(B)** By comparison of OTUs between treatments at 0 h, differences in absolute number of OTUs excluding Ishi-1 were identified at family or ^∗^higher rank. Remarkable reductions (<100 OTUs) are shown with red bars.

To identify the core bacterial components of CLas-associated microbiota influencing survival of CLas, we performed a metagenomic analysis using the oxytetracycline-treated and water-treated (0 h) communities. A total of 702 618 high-quality sequences and 9304 operational taxonomic units (OTUs) of bacteria (≥97% sequence identity of 16S rDNA sequences) were generated from the communities in both treatments. Comparative analysis revealed that oxytetracycline treatment typically reduced a subset of bacteria belonging to the classes *Actinobacteria*, *Flavobacteria*, and *Proteobacteria* (Figure 4A). By comparison of OTUs between treatments, we identified a sub-community which was susceptible to oxytetracycline (Figure 4B). This revealed remarkable reductions in the number of OTUs of the families *Comamonadaceae*, *Flavobacteriaceae*, *Microbacteriaceae*, and *Pseudomonadaceae*, which are members of the core bacterial community in CLas-infected citrus leaves (Zhang et al., 2013b; Blaustein et al., 2017). In contrast, those exceptional taxa were common in a CLas-free bacterial community from a healthy leaf (Supplementary Figure S10). This implies that CLas infection strongly influences alterations of the bacterial community structure. Overall, these findings show that oxytetracycline produced structural changes in the CLas-associated bacterial community that decreased the survival of CLas.

## Discussion

**FIGURE 5:**
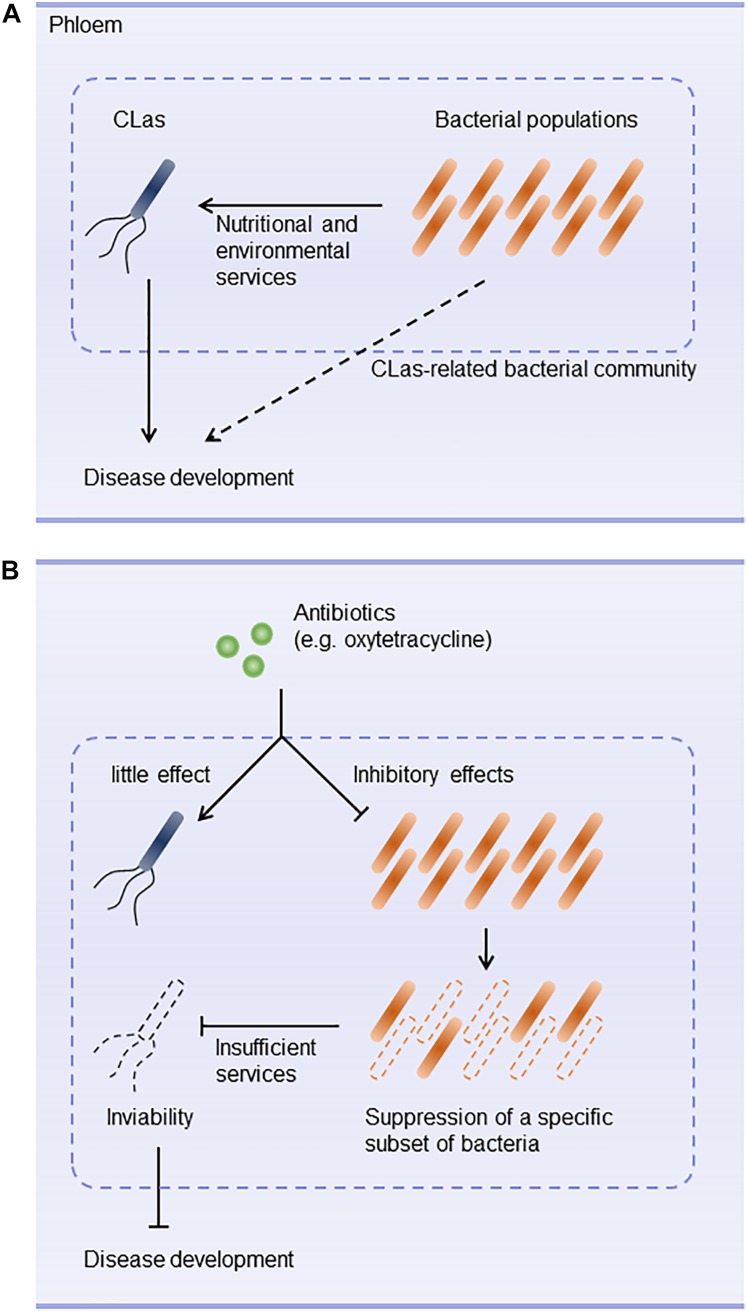
Hypothetical scenario of bacterial community ecology in development of huanglongbing. **(A)** Bacterial composition of CLas-associated microbiota plays a key role in nutritionally and environmentally supporting colonization of the host by CLas. Interplay between CLas and phloem-inhabiting bacteria constructs a community that is likely to contribute to development of huanglongbing. **(B)** Antibiotic stress triggers structural changes in the CLas-associated microbiota. For example, oxytetracycline does not inhibit CLas but eradicates a sub-community that supports CLas. Changes in the community structure make the community less habitable for CLas.

Huanglongbing is a devastating disease of citrus worldwide. Antibiotics such as oxytetracycline and β-lactams are used to control CLas (Zhang et al., 2013a,b; Hu and Wang, 2016). However, they don’t always eliminate CLas and are unlikely to accelerate recovery from CLas infection. These difficulties prompted us to hypothesize that antibiotics may not have direct effects on CLas. Our results demonstrate that CLas was not susceptible to antibiotics (except the high concentrations of polymyxin B) and was in fact promoted by oxytetracycline treatment. Furthermore, the survival of CLas was inhibited when the subset of CLas-associated microbiota was eliminated by oxytetracycline treatment *in vitro*. These results suggest that a specific subset of bacteria susceptible to oxytetracycline plays a critical role in providing ecological services that contribute to the survival of CLas in the phloem and to disease development (Figures 5A,B).

A major objective of this study was to investigate potential roles of CLas-associated microbiota in supplying ecological services for the survival of CLas. Microbiological factors influencing the viability of CLas are still unknown. The functional traits developed in CLas-associated microbiota could be explained by nutrient availability and biofilm formation. Nutrient availability is the most important factor in the establishment of habitability for CLas. CLas has a relatively small genome, associated with the loss of metabolic and regulatory capabilities (Duan et al., 2009; Katoh et al., 2014; Zheng et al., 2015). On the other hand, it conserves several genes encoding transporter isozymes, which play a significant role in acquiring sugars, amino acids, fatty acids, minerals, and vitamins present within the phloem (Duan et al., 2009). One would expect that such nutrients metabolized by the phloem-inhabiting bacteria could contribute to the availability of nutrients in the phloem (Singh et al., 2017). We are currently investigating possible bacterial metabolites that not only are uniquely produced in the CLas-associated microbiota, but also can be consumed by CLas. Associations between the community structure of the phloem-inhabiting bacteria and the ability to metabolize nutrients within the community will need to be elucidated in future work.

A biofilm is an assembly of a complex bacterial community that confers unique features (e.g., colonization, nutrient diffusion, protection from toxic substances, antimicrobial resistance, enhancement of virulence) that assist bacterial survival and persist within an environment (Burke et al., 2011; Cordero and Datta, 2016). Changes in starch accumulation in the phloem are typical in CLas-infected citrus trees (Schneider, 1968). The starch formed in infected leaves is morphologically similar but biochemically different from that in healthy leaves (Gonzalez et al., 2012). We assumed that the starch accumulation helps the survival of CLas and the development of CLas-associated microbiota in a similar way as in biofilms. A recent study has revealed that a CLas-specific effector, LasΔ5315, induced excessive starch accumulation in transient assays using *Nicotiana benthamiana* (Pitino et al., 2018). The CLas *luxR* gene encodes the LuxR protein, which is involved in cell-to-cell communication and needs to bind cognate N-acylhomoserine lactones for activity (Tsai and Winans, 2010), although CLas does not conserve genes involved in the biosynthesis of these lactones. These facts indicate that CLas triggers the alteration of the phloem environment and assembles phloem-inhabiting bacteria into CLas-associated microbiota though this specific communication process. The degradation of the CLas-associated microbiota could be a way to reduce the ecological services they provide for CLas, accelerating the elimination of CLas.

Application of antibiotics is effective in treating citrus greening disease. In this study, however, CLas growth was enhanced when exposed to oxytetracycline. The action of oxytetracycline as an enhancer rather than as an inhibitor *in vitro* is apparently inconsistent with the effective control of CLas by oxytetracycline in orchards (Hu and Wang, 2016). The reason for the enhancement of CLas growth due to oxytetracycline is still unclear. In the action of oxytetracycline, oxytetracycline acts both as a strong chelator of divalent metals and as a scavenger of reactive oxygen species (ROS) owing to the substituents on its aromatic ring (Griffin et al., 2010; Wang et al., 2016). In our preliminary test, oxytetracycline was unlikely to support CLas growth as a chelator while exogenous H_2_O_2_ supported the growth of Ishi-1 (data not shown). In addition, it has been highlighted that antibiotics act as signal molecules in bacteria and depending on the bacterial species, influence bacterial developmental program such as bacterial populations and stress responses (Ratcliff and Denison, 2011; Romero et al., 2011). Biofilm formation was induced by several antibiotics including aminoglycoside (a protein synthesis inhibitor), beta-lactam (a cell wall biosynthesis inhibitor), fluoroquinolone (a DNA gyrase and topoisomerase IV inhibitor), and tetracycline (a protein synthesis inhibitor) antibiotics at sub-inhibitory concentration (Bagge et al., 2004; Hoffman et al., 2005; Romero et al., 2011). In the case of the biofilm formation of *Staphylococcus intermedieus* due to beta-lactam, fluoroquinolone, and tetracycline antibiotics, the enhancement was likely to involve the LuxS/AI-2 signaling system which regulates cell-to cell communication (Ahmed et al., 2009). Also, macrolide (a protein synthesis inhibitor) and tetracycline antibiotics against *Pseudomonas aeruginosa* stimulated the type three secretion system (T3SS) and showed in consequence enhanced virulence of *P. aeruginosa* (Ader et al., 2005; Linares et al., 2006) An increase in the expression of the genes involved in exopolysaccharide production was observed in *Streptococci* when exposed to those (Rachid et al., 2000). In relation to this, self-produced pigments such as phenazine, antimicrobial and redox-active family of pigment, observed in *P. aeruginosa* triggered SoxR-regulated genes responsible for exopolysaccharide production whereas those genes were initially found to control gene expression regarding oxidative stresses (Dietrich et al., 2008). Antibiotics-driven interspecies interactions involved complex chemical interactions, resulting in structural and functional maturity of a microbial community (Keller and Surette, 2006; Romero et al., 2011). These studies suggest a complex role for antibiotics in regulating community behavior and cell-to-cell communication of CLas in the CLas-associated microbiota.

Culturing of unculturable bacteria provides a new insight into understanding microbiological features. Our KEGG genomic analysis has clarified the nature of CLas metabolism and offers opportunities to find candidate chemicals for media production. Our newly developed culture medium was successful for growing CLas *in vitro*. Our related work has cultured CLas from other citrus cultivars also (data not shown). These results allow the possibility to detect CLas from asymptomatic citrus. The development of a new detection technique will contribute to the control of CLas. On the other hand, enhancing the quality of the media has been the biggest challenge for us in achieving the formation of CLas colonies in culture. In addition to our analytical approach focusing on finding primary metabolites necessary for CLas, it will be essential to find secondary metabolites needed by CLas in culture because of its dependence on a host and other bacteria. To achieve the cell-to-cell communications described above, cell aggregation and adhesion of CLas might be stimulated by bacterial factors generated by co-culture with other bacteria (Stewart, 2012).

## Conclusion

Our study provides a partial explanation of how CLas-associated microbiota establish habitability for CLas. Ishi-1 was not susceptible to oxytetracycline *in vitro*, which is apparently inconsistent with the results of field trials. We speculate that oxytetracycline eliminated a particular sub-community of CLas-associated bacteria, thereby decreasing the survival of Ishi-1. This suggests that the presence of cohabiting bacteria is one of the key determinants for CLas establishment in the host. Dependence on the functions of CLas-cohabitating bacteria and perhaps other bacteria might be crucial for CLas to acquire nutritional and ecological services. It will be interesting to assess whether antibiotic-mediated alteration of community structures and functions is associated with virulence of CLas and the development of huanglongbing.

## Author Contributions

KF, TI, and TF conceived and designed the experiments. KF and TF contributed to the experiments, data analysis, and writing the manuscript.

## Conflict of Interest Statement

The authors declare one relevant Japanese patent application, 2017-025496 is associated with development of the new culture medium for CLas.

## Footnotes

1 http://www.genome.jp/kegg/
