# Supplementary material for: Alterations of Candidatus Liberibacter asiaticus-Associated Microbiota Decrease Survival of Ca. L. asiaticus in in vitro Assays
Source: Front Microbiol. 2018 Dec 21;9:3089. doi: 10.3389/fmicb.2018.03089 (PMC6308922; doi:10.3389/fmicb.2018.03089)
Supplement: Supplementary file 1 [file Data_Sheet_1.pdf]

Supplementary Material

Alterations of *Candidatus* Liberibacter asiaticus-associated microbiota decrease survival of *Ca.* L. asiaticus in *in vitro* assays

Kazuki Fujiwara, Toru Iwanami, and Takashi Fujikawa*

*** Correspondence:** Takashi Fujikawa, ftakashi@affrc.go.jp

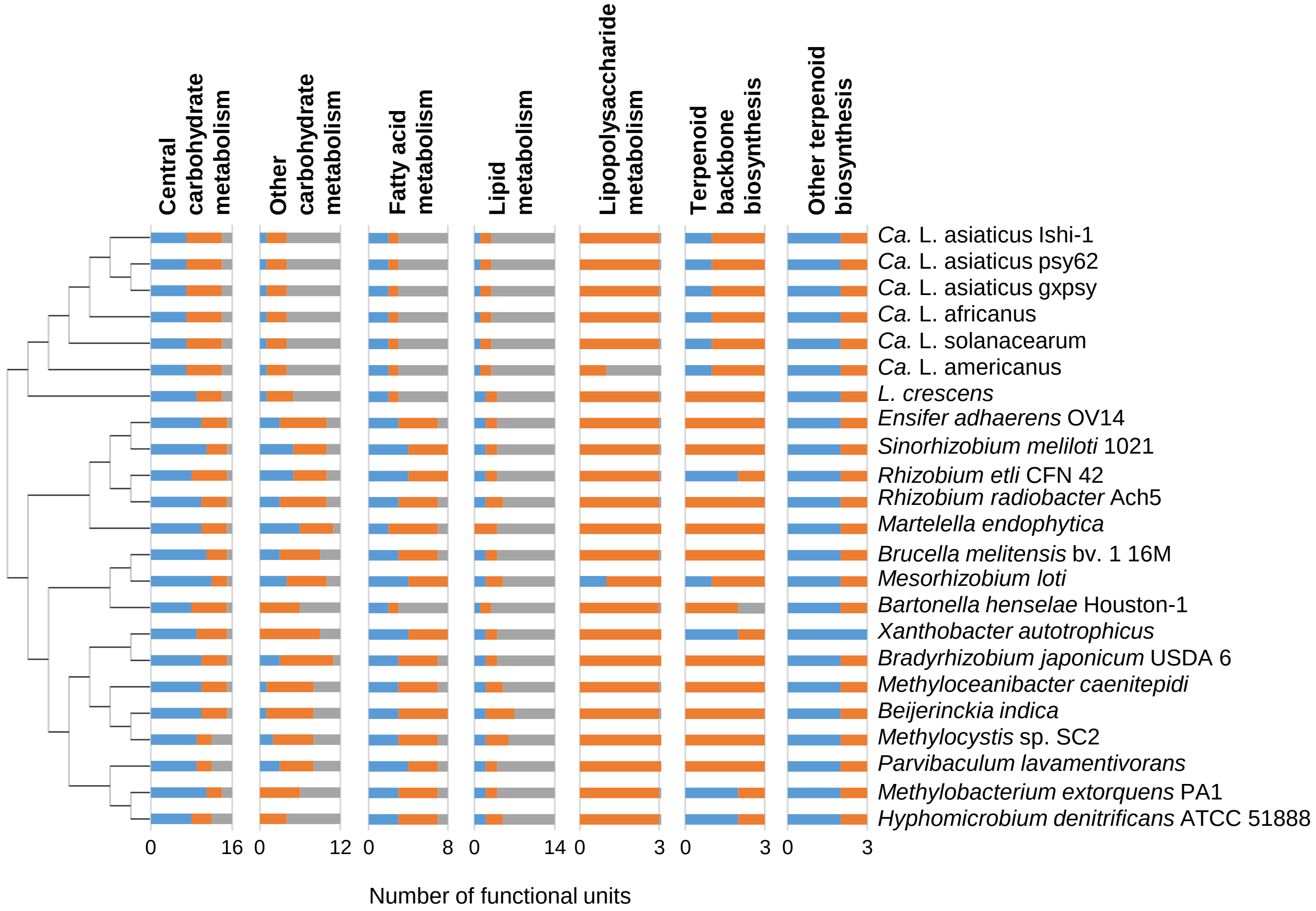

Central carbohydrate metabolism
Other carbohydrate metabolism
Fatty acid metabolism
Lipid metabolism
Lipopolysaccharide metabolism
Terpenoid backbone biosynthesis
Other terpenoid biosynthesis
Ca. L. asiaticus Ishi-1
Ca. L. asiaticus psy62
Ca. L. asiaticus gxpsy
Ca. L. africanus
Ca. L. solanacearum
Ca. L. americanus
L. crescens
Ensifer adhaerens OV14
Sinorhizobium meliloti 1021
Rhizobium etli CFN 42
Rhizobium radiobacter Ach5
Martelella endophytica
Brucella melitensis bv. 1 16M
Mesorhizobium loti
Bartonella henselae Houston-1
Xanthobacter autotrophicus
Bradyrhizobium japonicum USDA 6
Methyloceanibacter caenitepidi
Beijerinckia indica
Methylocystis sp. SC2
Parvibaculum lavamentivorans
Methylobacterium extorquens PA1
Hyphomicrobium denitrificans ATCC 51888
0
16
0
12
0
8
0
14
0
3
0
3
0
3
Number of functional units

Supplementary Figure S1 | Carbohydrate and lipid metabolism pathway analysis of 23 species of α-Proteobacteria. Blue, full functional unit; orange, partial functional unit; grey, no functional unit. Phylogenetic relationships were analysed on the basis of nucleotide sequences of 16S ribosomal DNA from GenBank by the neighbour-joining method in MEGA 6 software. Stability was assessed using 1000 bootstrap replications.

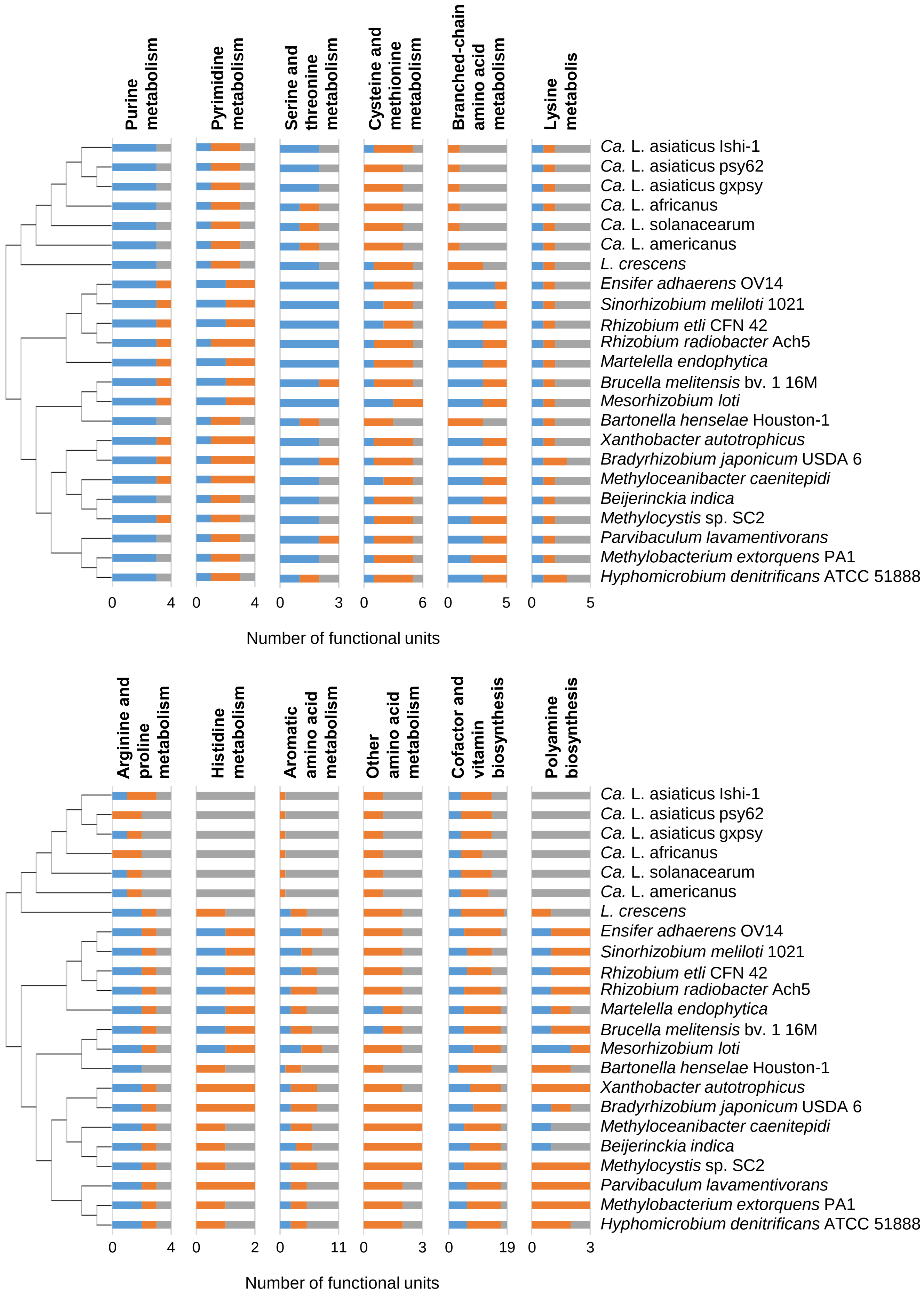

Purine metabolism
Pyrimidine metabolism
Serine and threonine metabolism
Cysteine and methionine metabolism
Branched-chain amino acid metabolism
Lysine metabolis
Ca. L. asiaticus Ishi-1
Ca. L. asiaticus psy62
Ca. L. asiaticus gxpsy
Ca. L. africanus
Ca. L. solanacearum
Ca. L. americanus
L. crescens
Ensifer adhaerens OV14
Sinorhizobium meliloti 1021
Rhizobium etli CFN 42
Rhizobium radiobacter Ach5
Martelella endophytica
Brucella melitensis bv. 1 16M
Mesorhizobium loti
Bartonella henselae Houston-1
Xanthobacter autotrophicus
Bradyrhizobium japonicum USDA 6
Methyloceanibacter caenitepidi
Beijerinckia indica
Methylocystis sp. SC2
Parvibaculum lavamentivorans
Methylobacterium extorquens PA1
Hyphomicrobium denitrificans ATCC 51888
0 4 0 4 0 3 0 6 0 5 0 5
Number of functional units
Arginine and proline metabolism
Histidine metabolism
Aromatic amino acid metabolism
Other amino acid metabolism
Cofactor and vitamin biosynthesis
Polyamine biosynthesis
0 4 0 2 0 11 0 3 0 19 0 3
Number of functional units

Supplementary Figure S2 | Amino acid and nucleotide metabolism pathway analysis of 23 species of α-Proteobacteria. Blue, full functional unit; orange, partial functional unit; grey, no functional unit. Phylogenetic relationships were analysed on the basis of nucleotide sequences of 16S ribosomal DNA from GenBank by the neighbour-joining method in MEGA 6 software. Stability was assessed using 1000 bootstrap replications.

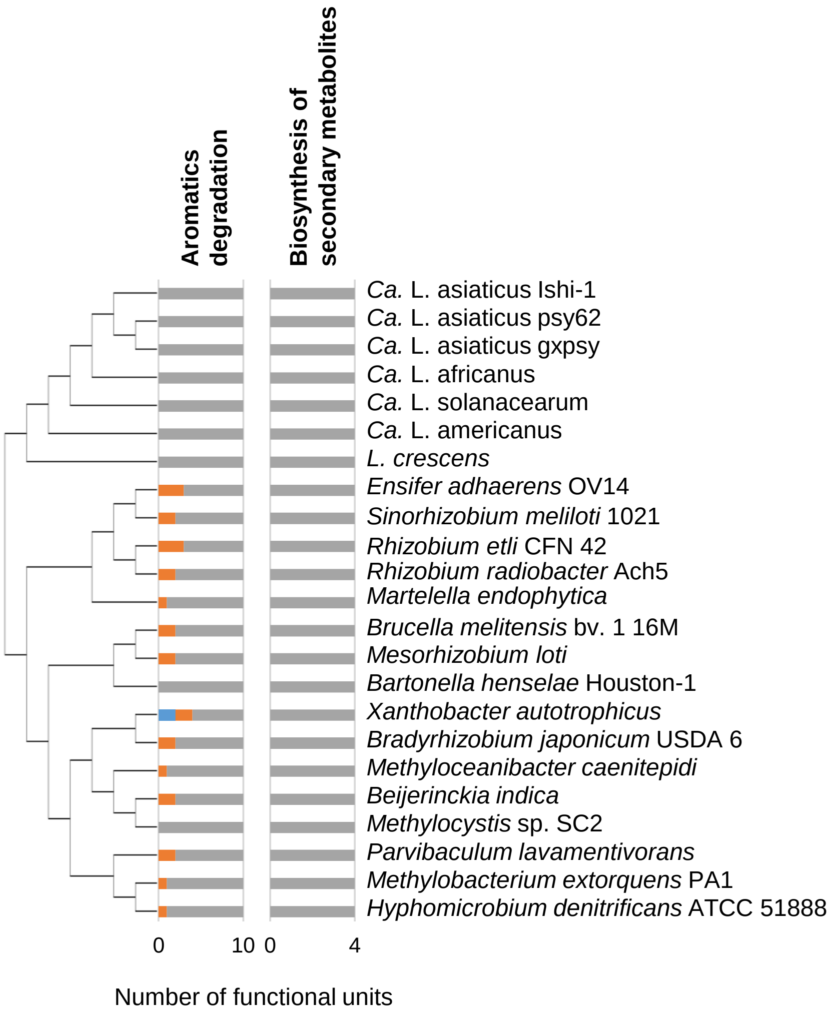

Aromatics degradation
Biosynthesis of secondary metabolites
Ca. L. asiaticus Ishi-1
Ca. L. asiaticus psy62
Ca. L. asiaticus gxpsy
Ca. L. africanus
Ca. L. solanacearum
Ca. L. americanus
L. crescens
Ensifer adhaerens OV14
Sinorhizobium meliloti 1021
Rhizobium etli CFN 42
Rhizobium radiobacter Ach5
Martelella endophytica
Brucella melitensis bv. 1 16M
Mesorhizobium loti
Bartonella henselae Houston-1
Xanthobacter autotrophicus
Bradyrhizobium japonicum USDA 6
Methyloceanibacter caenitepidi
Beijerinckia indica
Methylocystis sp. SC2
Parvibaculum lavamentivorans
Methylobacterium extorquens PA1
Hyphomicrobium denitrificans ATCC 51888
0
10
0
4
Number of functional units

Supplementary Figure S3 | Secondary metabolism pathway analysis of 23 species of α-Proteobacteria. Blue, full functional unit; orange, partial functional unit; grey, no functional unit. Phylogenetic relationships were analysed on the basis of nucleotide sequences of 16S ribosomal DNA from GenBank by the neighbour-joining method in MEGA 6 software. Stability was assessed using 1000 bootstrap replications.

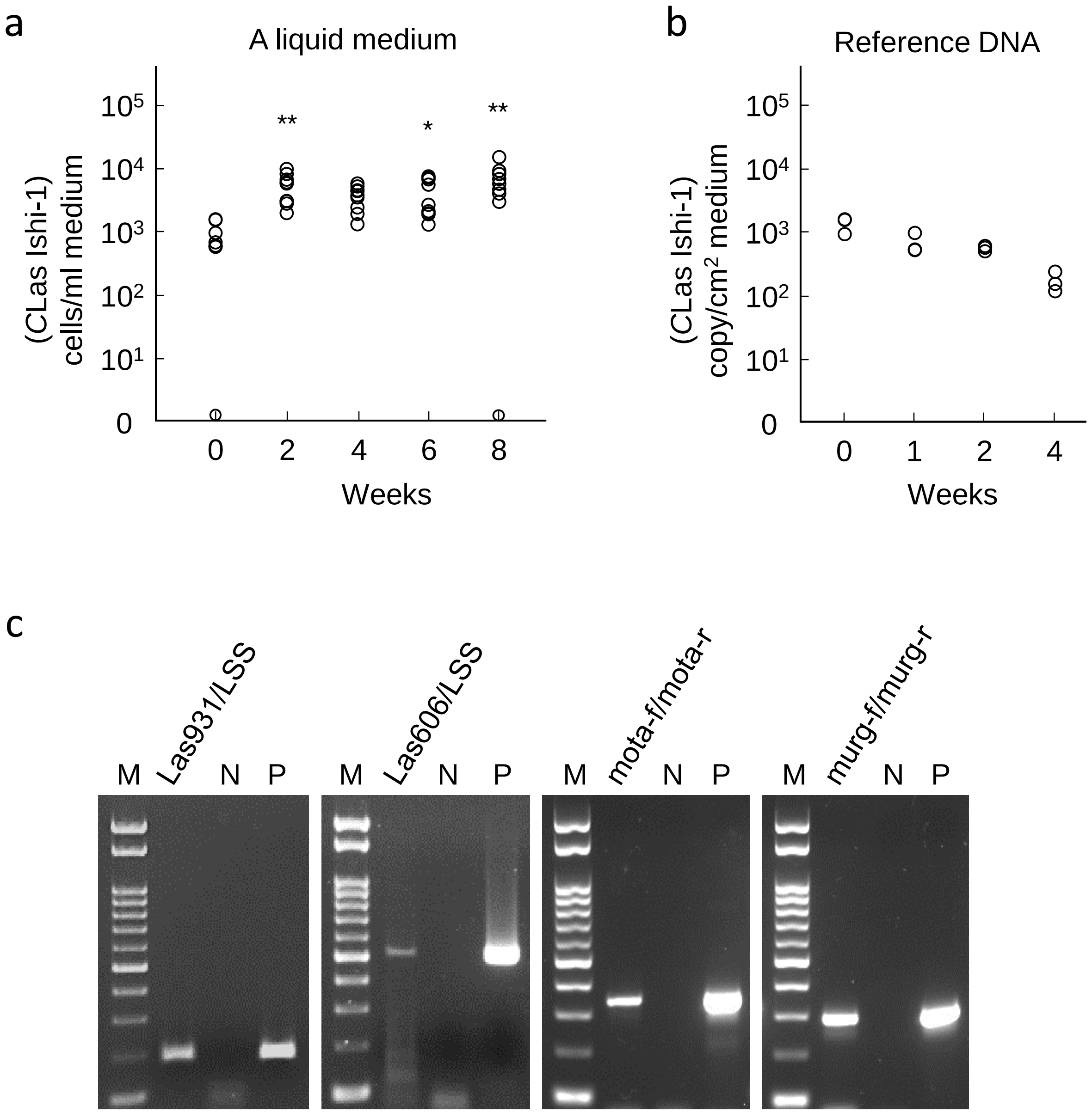
a
A liquid medium
cells/ml medium
(CLas Ishi-1)
10^5
10^4
10^3
10^2
10^1
0
**
*
**
0
2
4
6
8
Weeks
b
Reference DNA
copy/cm^2 medium
(CLas Ishi-1)
10^5
10^4
10^3
10^2
10^1
0
0
1
2
4
Weeks
c
M
Las931/LSS
N
P
M
Las606/LSS
N
P
M
mota-f/mota-r
N
P
M
murg-f/murg-r
N
P

Supplementary Figure S4 | *In vitro* culture status of CLas Ishi-1. (A) Liquid culture medium supplemented with ampicillin (50 ppm) promoted proliferation of Ishi-1 without contamination. Ishi-1 was quantified by real-time PCR using DNA templates prepared from nine replicates at each time point. Statistical significance was determined in comparison with concentration at 0 weeks by Dunnett's test (P < *0.05, **0.01) in R statistical software. **(B)** Reference DNA of Ishi-1 decreased on culture plate over 4 weeks. Reference DNA was quantified by real-time PCR using DNA templates prepared from 1-cm^2 pieces of agar (n = 3, each value plotted individually). **c,** Ishi-1 was detectable in conventional PCR assays with several candidate primers: Las931/LSS (for 16S rRNA genes, 195 bp), Las606/LSS (for 16S rRNA genes, 501 bp), mota-f/mota-r (for flagellar motor protein gene *motA*, 329 bp), and murg-f/murg-r (for lipid II flippase gene *murG*, 191 bp). M: marker; N: negative control; P: positive control.

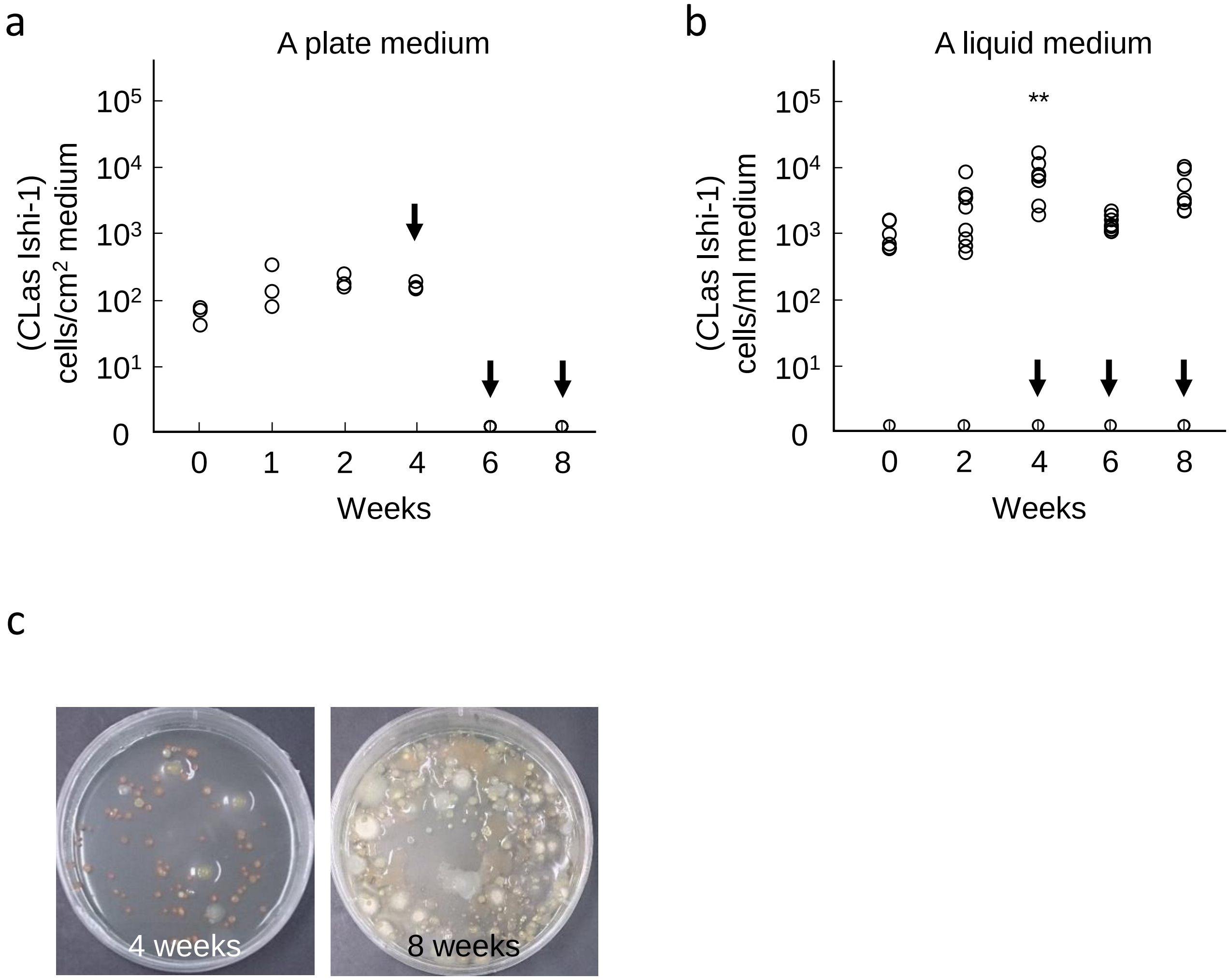
a
A plate medium
(CLas Ishi-1) cells/cm2 medium
10^5
10^4
10^3
10^2
10^1
0
0 1 2 4 6 8
Weeks
b
A liquid medium
(CLas Ishi-1) cells/ml medium
**
0 2 4 6 8
Weeks
c
4 weeks
8 weeks

Supplementary Figure S5 | Contamination of *in vitro* culture of CLas Ishi-1. (A) Culture of CLas Ishi-1 in a plate medium without ampicillin. **(B)** Culture of CLas Ishi-1 in a liquid medium without ampicillin. Contamination (arrows) prevented Ishi-1 from growing on plates and in liquid media without ampicillin (50 ppm). In plate culture, contamination appeared at 2 weeks of incubation but did not inhibit CLas growth then; but by 4 weeks it produced some yellowish discoloration of the culture plate, resulting in severe contamination afterwards. In liquid culture, contamination appeared in one of nine replicates at 4 weeks. Ishi-1 was quantified by real-time PCR using DNA templates prepared from 1-cm^2 pieces of agar (n = 3, each value plotted individually) and nine replicates of liquid culture at each time point. Data of plate medium at 2 weeks are used in Figure 2A. Statistical significance was determined in comparison with concentration of CLas at 0 weeks by Dunnett's test (P < **0.01) in R statistical software. **(C)** Contamination indicated by arrows in **(A)**.

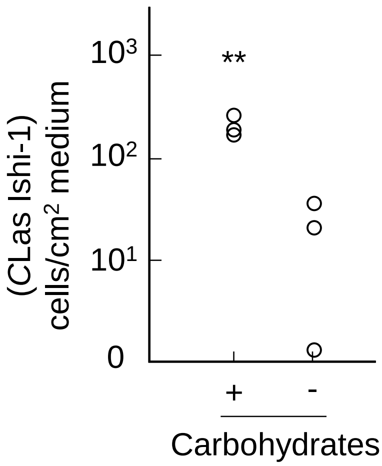

cells/cm2 medium
(CLas Ishi-1)
10³
10²
10¹
0
**
+
-
Carbohydrates

Supplementary Figure S6 | Carbohydrates (glucose, sucrose, fructose, and starch) are essential for growth of Ishi-1 on culture medium. Data of plate culture at 2 weeks shown in Figure 2A (Ctrl) are used for carbohydrates +. Ishi-1 was quantified by real-time PCR using DNA templates prepared from 1-cm^2 pieces of agar ($n = 3$, each value plotted individually). Significance was determined by Student's *t*-test ($P <$ **0.01) in R statistical software.

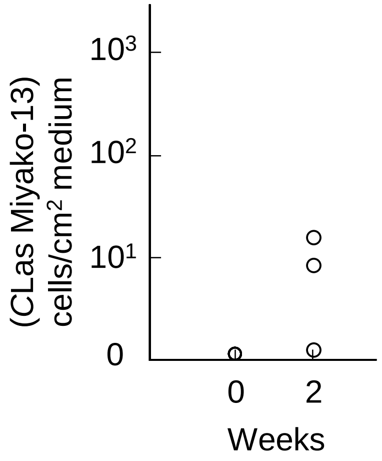

(CLas Miyako-13)
cells/cm2 medium
10^3
10^2
10^1
0
0
2
Weeks

Supplementary Figure S7 | CLas Miyako-13 grew poorly in plate culture. CLas Miyako-13 struggled to grow on plate medium, in contrast to Ishi-1 at 2 weeks (Figure 2A, Ctrl). Culture procedures were the same as for Ishi-1. Miyako-13 was quantified by real-time PCR using DNA templates prepared from 1-cm^2 pieces of agar (*n* = 3, each value plotted individually). Culture samples were collected at 0 and 2 weeks' incubation.

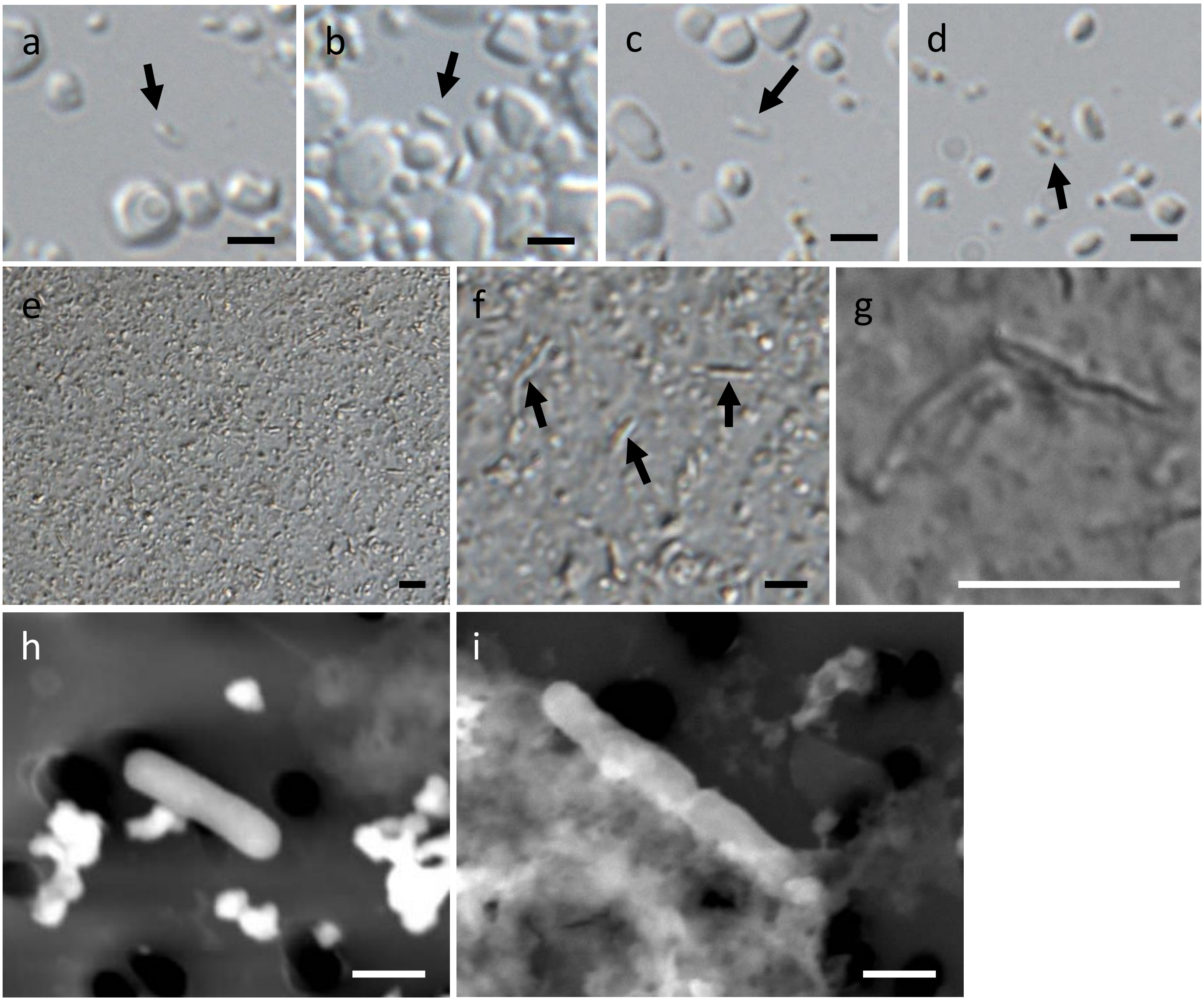
a
b
c
d
e
f
g
h
i

Supplementary Figure S8 | Microscopic observations of CLas in plate and liquid culture. (A–D) Survival of CLas-like cells in plate culture (arrows) and plant-derived substances of various sizes at 2 weeks. Bars = 5 μm. **(E–G)** Elongated CLas-like cells in liquid culture at 4 weeks. Bars = 10 μm. **(H–I),** SEM observations of single and elongated CLas-like cells in liquid culture at 4 weeks. Bars for **(H)** and **(I)** = 1 μm.

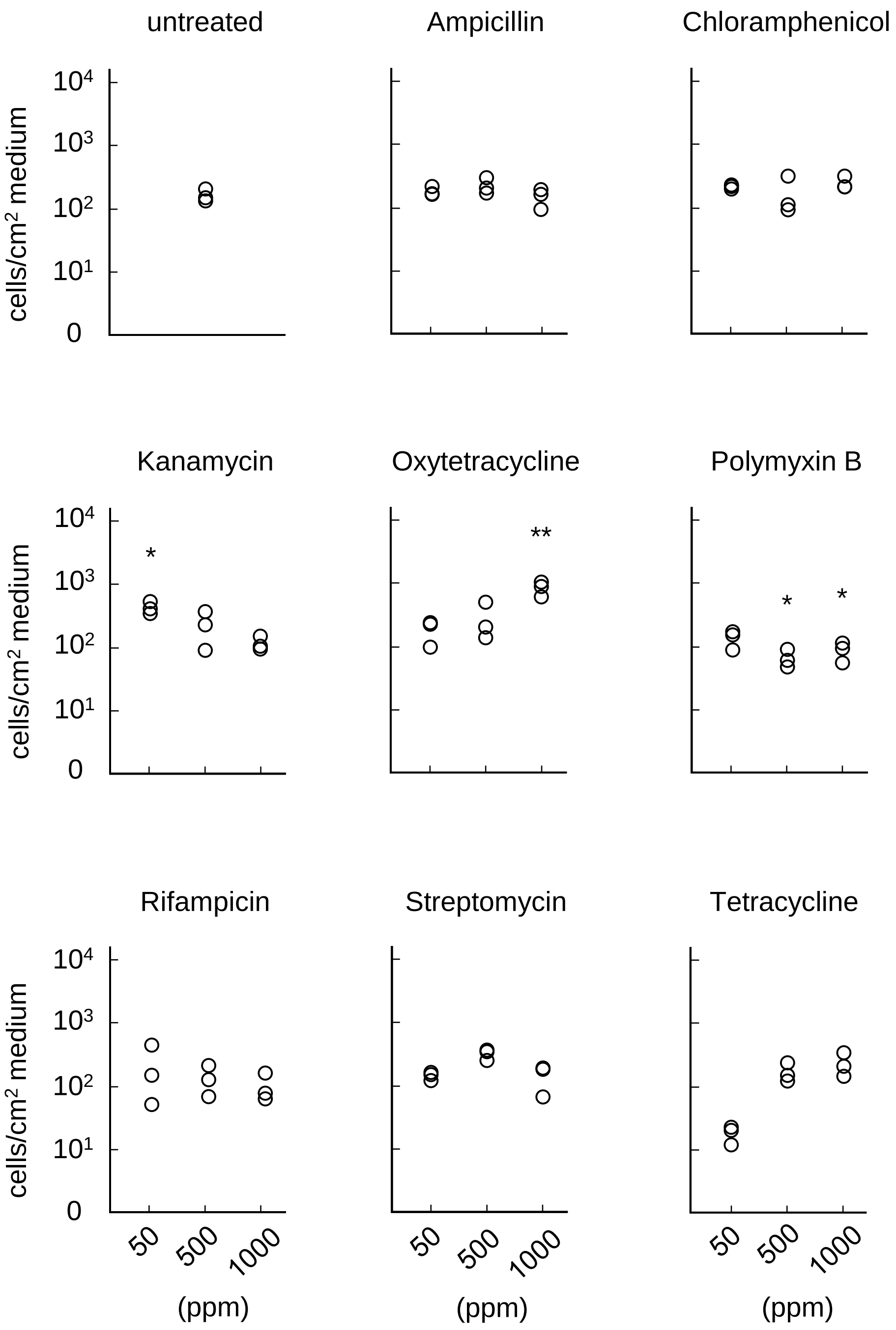

untreated
Ampicillin
Chloramphenicol
Kanamycin
Oxytetracycline
Polymyxin B
Rifampicin
Streptomycin
Tetracycline
cells/cm2 medium
10⁴
10³
10²
10¹
0
*
**
50
500
1000
(ppm)

Supplementary Figure S9 | *In vitro* antibiotic assays. Results show responses of CLas Ishi-1 to eight antibiotics at 2 weeks. Results of oxytetracycline (1000 ppm) were significantly different ($P <$ **0.01) and results of kanamycin (50 ppm), and polymyxin B (500 and 1000 ppm) were significantly different ($P <$ *0.05) from those of untreated cells (Figure 2A). Ishi-1 was quantified by real-time PCR using DNA templates prepared from 1-cm^2 pieces of agar (n = 3, each value plotted individually). Significance was determined with Dunnett's test in R statistical software.

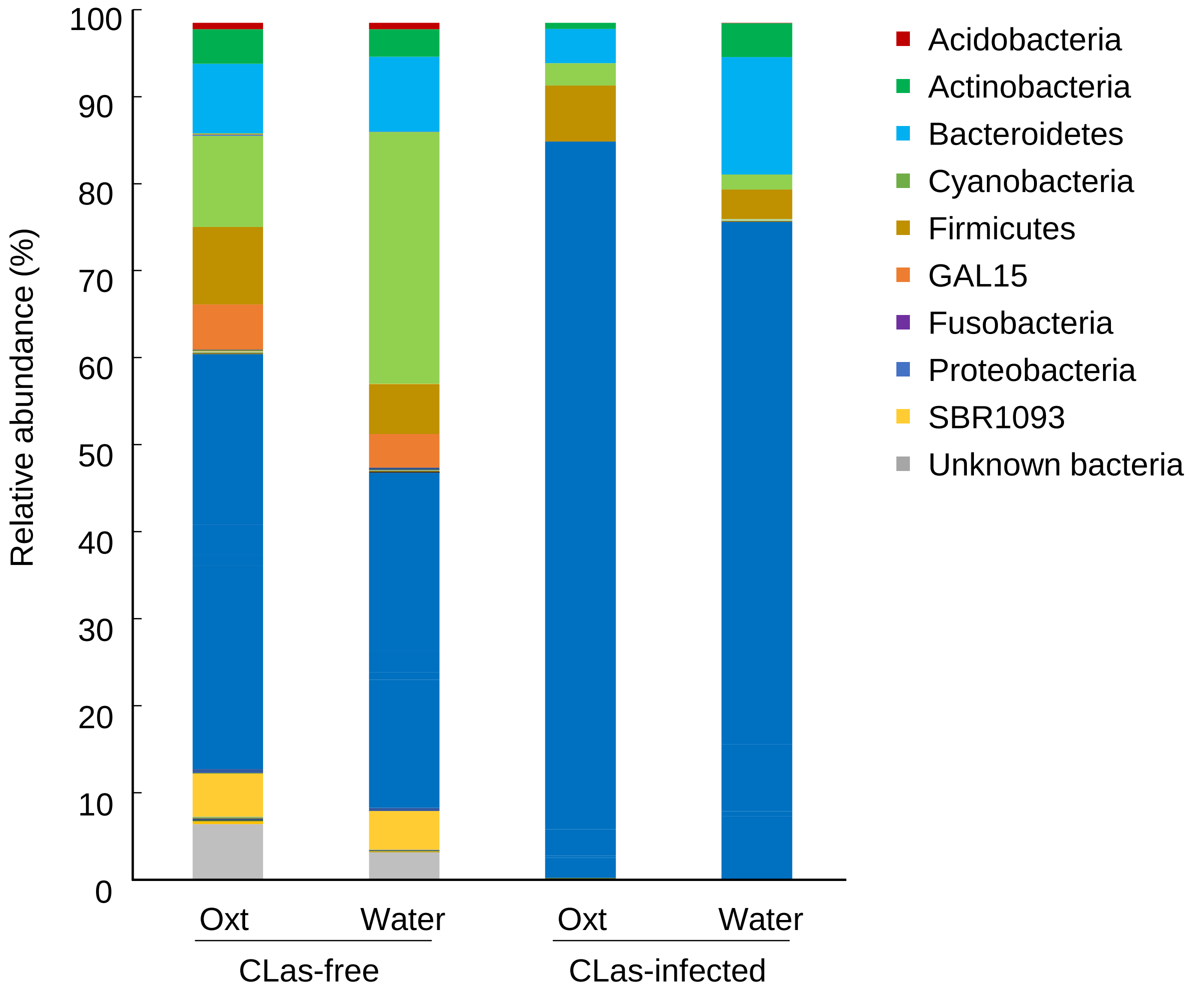
100
90
80
70
60
50
40
30
20
10
0
Relative abundance (%)
Oxt
Water
Oxt
Water
CLas-free
CLas-infected
Acidobacteria
Actinobacteria
Bacteroidetes
Cyanobacteria
Firmicutes
GAL15
Fusobacteria
Proteobacteria
SBR1093
Unknown bacteria

Supplementary Figure S10 | Metagenomic characterization of CLas-free and CLas-associated bacterial communities in oxytetracycline and water treatments. Representative samples were prepared by mixing three replicates of DNA extracted from each extract. The CLas-free (healthy) bacterial communities in both treatments (0 h) yielded 343 600 high-quality sequences, resulting in 4165 operational taxonomic units (≥97% sequence identity of 16S rDNA sequences). CLas-infected bacterial communities were provided by the metagenomic data shown in Figure 4. Among the 27 phyla identified, 9 predominated.

Supplementary Table S1. Chemical composition of new culture medium for CLas Ishi-1.

Substance	Manufacturer	Quantity (g/100 mL)
Phytone	BD Difco	0.5
Yeast extract	BD Difco	0.2
Glucose	Wako	0.1
Fructose	Wako	0.1
Sucrose	Wako	0.1
Starch	Wako	0.1
Ketoglutarate	Sigma	0.1
MEM amino acids essential (50×)	Wako	0.5*
MEM amino acids non-essential (100×)	Wako	1*
Glutamine	Wako	0.1
Methionine	Wako	0.1
Cysteine	Wako	0.1
Cystine	Wako	0.1
Sodium glycerophosphate	Wako	0.5
CoA	Wako	<0.1
Glycerol	Wako	1*
Bromothymol blue	Wako	0.001
Agarose (or gellan gum)	Wako	2 (1)

*mL.

Supplementary Table S2. Primers and probes used in this study.

Name	Sequence
Las606	5′-GGA GAG GTG AGT GGA ATT CCG A-3′
LSS	5′- ACC CAA CAT CTA GGT AAA AAC C-3′
Las931	5′-CAG CCC TTG ACA TGT ATA GGA CG-3′
HLBas	5′-TCG AGC GCG TAT GCA ATA CG-3′
HLBr	5′-GCG TTA TCC CGT ACA AAA AGG TAG-3′
HLBp	5′-AGA CGG GTG AGT AAC GCG-3′
mota-f	5′-ATA CAA AGC ATT GGG GCA AG-3′
mota-r	5′-CAA TGG CAG GTA AGG ATT CG-3′
murg-f	5′-CAG AGA TGC AGC GTA AAC GA-3′
murg-r	5′-TAG CTC CCC CTC CTT CTT GT-3′

Supplementary Table S3. Genes differentially expressed on exposure to oxytetracycline.

Gene	Description	*P*-value[a]	Upregulation ratio (oxytetracycline)
recJ	ssDNA exonuclease	0.003	11.84
CGUJ_01130	proline/glycine betaine ABC transporter permease	0.01	10.47
CGUJ_04070	oligoendopeptidase F	0.003	11.84

a. Statistical significance was determined by Student's *t*-test.

Supplementary Table S4. Upregulation of genes responsible for membrane transport and drug resistance under oxytetracycline treatment relative to control treatment.

Gene	Description	KEGG pathway	Upregulation ratio (oxytetracycline)
dnaK	Molecular chaperone DnaK	Membrane transport	4.03
omp	Surface antigen (D15)	Membrane transport	3.78
pstC	ABC transporter membrane-spanning protein	Membrane transport	4.39
thiQ	Thiamine transporter ATP-binding subunit	Membrane transport	5.43
CGUJ_01130	Proline/glycine betaine ABC transporter permease	Membrane transport	10.47*
CGUJ_02130	ABC transporter membrane-spanning protein	Membrane transport	8.95
CGUJ_03055	Component of type IV pilus	Membrane transport	8.13
CGUJ_05380	Ascorbate-specific PTS system enzyme IIC/IIB	Membrane transport	4.58
glyA	Serine hydroxymethyltransferase	Drug resistance	4.58
mraY	Phospho-N-acetylmuramoyl-pentapeptide-transferase	Drug resistance	6.36
mrcA	Penicillin-binding peptidoglycan synthetase protein	Drug resistance	3.72
murG	N-acetylglucosaminyl transferase	Drug resistance	4.80
CGUJ_02935	Serine protease DO-like protease	Drug resistance	4.41
CGUJ_04275	Thymidylate synthase	Drug resistance	5.26

*Gene expression was significantly different from control ($P < 0.05$; Student's *t*-test).
